# Dynamics of a microbial community during an effective boost MEOR trial using high-throughput sequencing

**DOI:** 10.1039/c7ra12245d

**Published:** 2018-01-02

**Authors:** Sanbao Su, Hao Dong, Lujun Chai, Xiaotao Zhang, Ibrahim M. Banat, Zhengliang Wang, Dujie Hou, Fan Zhang, Yuehui She

**Affiliations:** School of Petroleum Engineering, Yangtze University Wuhan Hubei 430010 China; College of Chemistry and Environmental Engineering, Yangtze University Jingzhou Hubei 434023 China sheyuehui@163.com; The Key Laboratory of Marine Reservoir Evolution and Hydrocarbon Accumulation Mechanism, Ministry of Education, School of Energy Resources, China University of Geosciences (Beijing) Beijing 100083 China fanzhang123@126.com; Faculty of Life and Health Sciences, University of Ulster Coleraine BT52 1SA UK

## Abstract

Using 454 pyrosequencing of 16S rRNA gene amplicons, microbial communities in samples of injection water and production water during a serial microbial enhanced oil recovery (MEOR) field trial in a water flooded high pour point oil reservoir were determined. There was a close microbial community compositional relationship between the injection water and the successful first round MEOR processed oil reservoir which was indicated by the result of 43 shared dominant operational taxonomic units detected in both the injection water and the production water. Alterations of microbial community after the injection of boost nutrients showed that microbes giving positive responses were mainly those belonging to the genera of *Comamonas*, *Brevundimonas*, *Azospirillum*, *Achromobacter*, *Pseudomonas*, and *Hyphomonas*, which were detected both in the injection water and in the production water and usually detected in oil reservoir environments or associated with hydrocarbon degradation. Additionally, microbes only dominant in the production waters were significantly inhibited with a sharp decline in their relative abundance. Based on these findings, a suggestion of re-optimization of the boost nutrients, targetting the microbes co-existing in the injection water and the oil reservoir and having survival ability in both surface and subsurface environments, rather than simple repeats for the subsequent *in situ* MEOR applications was proposed.

## Abstract 2

The microbes stimulated during injection of boost nutrients were mainly those detected both in injection water and production water.

## Graphical abstract

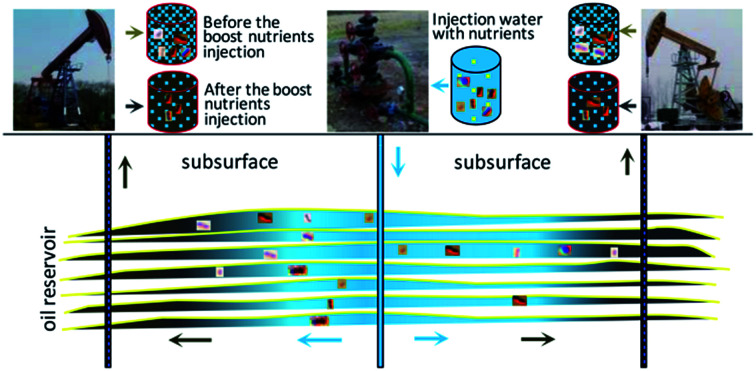

## Introduction

It is a well-established fact that oil reservoirs harbor and sustain diverse microbial communities. Natural untouched oil reservoirs usually have low redox potentials and limited electron donors and acceptors, and only strict anaerobes can normally survive, be active and be truly considered indigenous in them.^1^ In practice, microbial populations of oil reservoirs are usually disturbed and significantly altered during the production process,^2^ especially during the microbial enhanced oil recovery (MEOR) process with nutrient injection for *in situ* stimulation of microbes. These activities invariably result in the introduction of some exogenous microbes and disappearance of some indigenous communities. It is not, therefore, surprising to detect mostly facultative and aerobic microbes in samples from oil reservoirs.^3–7^ In a recent investigation of microbial ecology of a water flooded oil reservoir, it was concluded that microorganisms present in the injection water have major effects on the microbial compositions of oil reservoirs.^6^ It is important to note, however, that sequencing data of shared operational taxonomic units (OTU) supporting this opinion was not substantial. Furthermore, it has been reported that only two out of 54 bacterial OTU in the injection water appeared in the well production waters.^5^

Knowledge of microbes inhabiting oil reservoirs is important for understanding the microbial ecology of oil reservoirs and the efficient applications involving augmentation or utilization the active microbes in oil production applications. Potential microbial activities such as hydrocarbon metabolism, biomass and biofilm formation and productions of acids, solvents, gases, bio-surfactants, biopolymers and emulsifiers can all be utilized in the oil recovery processes.^2,8–11^ The MEOR processes have therefore been suggested to be an important tertiary oil recovery mechanism.^12^ Three main strategies are mainly used in MEOR field trials including: (1) nutrient injection to stimulate *in situ* microorganisms inhabiting in oil reservoirs, (2) augmentation with a selected microbial culture, and (3) injection of *ex situ* microbial products.^2,13,14^ It is important to identify changes in microbial community structure to reveal how the process is working under whatever strategy is applied and to gain further knowledge for optimization of such applications. Molecular biological techniques of denaturing gradient gel electrophoresis and use of a clone library have all been reported for monitoring changes in microbial communities during MEOR field trials.^9,11,15,16^

The microbial communities can be determined using high-throughput sequencing technology. Therefore, in this study this technique was used to determine the microbes inhabiting the Liaohe oil field reservoir (China) during a serial MEOR field trial. The two main objectives were: (1) to reveal the microbial relationships between the injection water and the high pour point oil reservoir experienced successful MEOR process with the first round injection of nutrients for *in situ* microbe stimulation, and (2) to identify the positive microbial groups that responded to the injection of boost nutrients. To achieve this, 454 pyrosequencing of 16S rRNA gene amplicons was used as a powerful tool to establish the dynamic alterations of the microbial community occurring during an effective boost MEOR trial.

## Material and methods

### Sample collection

A serial *in situ* MEOR process was carried out in the J68-50 well group which includes one injection well (IW) and seven production wells in the Liaohe Oil Field (China). The oil production from the oil reservoir commenced in 1986, and in October 1986, water flooding was started. The first round of MEOR field trials was carried out from December 30th, 2010 to January 1st, 2011, in which a total of 500 m^3^ of nutrients were injected. Microbiological monitoring details and oil well group production characteristics were reported in a previous paper.^11^ After five months, the second round of MEOR field trial was carried out. A total of 502 m^3^ of nutrients containing (1 L) of 20 g corn starch powder, 1.6 g of diammonium phosphate [(NH_4_)_2_HPO_4_], and 0.2 g of potassium nitrate (KNO_3_) were injected between May 11th and May 16th, 2011.

According to previous findings, the microbial communities of five production well samples (J69-151C, J67-551, J68-552, J69-349, J70-050C) collected at two different times after the first round of nutrient injections clustered into two different groups.^11^ The aim of the present research was to monitor the dynamics of microbial communities during an effective boost MEOR application in order to explore the relationship of microbes present in the production wells and in the injection well for an oil reservoir which had experienced a first round nutrient injection for *in situ* MEOR application. Two production wells: J70-050C (PW1) and J68-552 (PW2) were chosen for sampling. Before this round of field trials (May 6th, 2011), water samples were collected from the J68-50 injection well and mixed oil/water liquids from two production wells J70-050C and J68-552. After the oil production increase appeared (June 14th, 2011), mixed oil/water liquids were collected from the same two production wells and labelled as PW1T and PW2T (T means treatment). All the samples were collected in sterile plastic bottles and transported immediately to the laboratory for molecular analyses.

### DNA extraction

Approximately 150–200 ml of mixed oil/water liquid sample was centrifuged at 10 000*g* for 10 min to pellet the cells. Genomic DNA was extracted from the collected cells following the manufacturer's protocol for the FastDNA SPIN Kit for Soil DNA Extraction (Qbiogene, Carlsbad, CA, USA).^9^ For each sample, DNA was extracted in duplicate to avoid bias, and then the samples were pooled together for the following analysis. The DNA obtained was purified using an Agarose Gel DNA Purification Kit (TianGen Biotech, Beijing, China) and stored at −20 °C.

### PCR amplification, pooling and pyrosequencing

To identify microbes present in each sample, 454 pyrosequencing of the 16S rRNA gene were carried out. A region approximately 526 bp covering the V1–V3 region of 16S rRNA gene in each sample was amplified using the 27F and 533R primers containing the A and B adaptors (454 Life Sciences).^17,18^ The reverse primer (A-533R) had an A adaptor with a ten base sample unique barcode sequence.

Polymerase chain reaction (PCRs) were carried out in triplicate in a 50 μl reaction system containing 0.6 μM each of the primer, approximately 5 ng of the template DNA, 1× PCR buffer, 2.5 U of Pfu DNA polymerase (MBI Fermentas, USA). Negative controls were performed without addition of the template DNA. The amplification program is described elsewhere.^18^ After amplification, the PCR products of the same sample were pooled and purified using a DNA gel extraction kit (Axygen, China).

Next, the concentration of each PCR product was determined and the quality was controlled.^18^ The working pool was a mixture of an equi-molar ratio of each amplicon which was subjected to emulsion PCR to generate amplicon libraries, as recommended by 454 Life Sciences. Amplicon pyrosequencing was performed from the A-end using 454/Roche A sequencing primer kit on a Roche Genome Sequencer GS FLX Titanium platform (Majorbio Bio-Pharm Technology Co., Ltd, Shanghai, China).

### Statistical and bioinformatics analysis

The raw multiplexed sequence reads were processed using QIIME-1.6.0 pipeline (http://www.qiime.org).^17^ During quality filtering, any low quality or ambiguous readout was removed. Then, the sequences were clustered, by default, into OTUs with 97% similarity. The representative sequences of each OTU were assigned using a RDP classifier against the Greengenes database.

Excel was used as a statistical tool using the AutoFilter and Pivot Table functions. Notably, in this step, the data related to OTUs with sequences failed in alignment and unclassified bacteria were removed. Shared OTU analysis was calculated using the VennDiagram package in R (version 2.15.3) (http://www.r-project.org).^19^ The hierarchy of columns in the heat map was based on the Bray–Curtis similarity and used the complete linkage in the R package gplots. Rarefaction curves were obtained using Analytic Rarefaction software (http://www.uga.edu/strata/software/Software.html) in which the expected number of OTUs *versus* the number of reads in each library were calculated.^6^ In this study, too many valid representative OTUs were obtained to construct a normal phylogenetic tree, and technical reproducibility thresholds were determined to conclude that OTUs defined by ≥10 reads in any sample are “dominant OTUs”. Sequences of dominant OTUs were used to construct a phylogenetic tree using the neighbor-joining method and to identify stimulated microbes.^9,20^

## Results

**Fig. 1:**
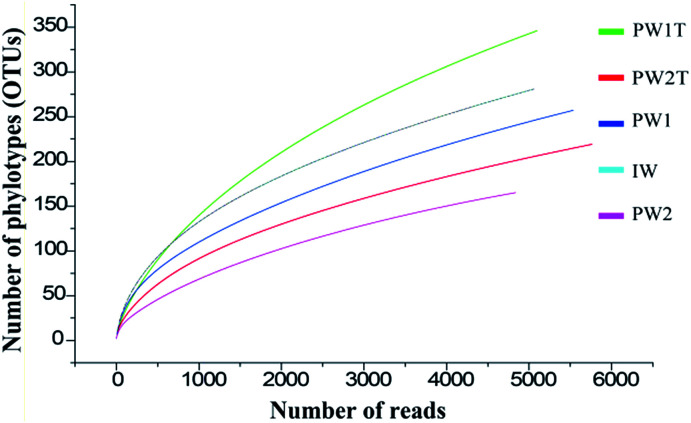
Rarefaction analysis of the samples of IW, PW1, PW1T, PW2 and PW2T. Numbers of OTUs and reads clustered by default at the 97% sequence identity using QIIME, and the rarefaction analysis used Analytic Rarefaction software.

A total of 26 307 valid reads were obtained, and were clustered into 984 OTUs that were arranged into bacteria from the five samples (IW, PW1, PW1T, PW2 and PW2T) using the 454 pyrosequencing and bioinformatic analysis results. The entire set of the raw reads is available at NCBI Sequence Read Archive under accession number of SRR826472. The numbers of reads in each sample ranged from 4838 to 5763, with OTUs ranging from 165 to 346. The rarefaction curves tended to approach the saturation plateau (Fig. 1).

According to the reproducibility thresholds determined, 155 OTUs out of 984 were grouped as dominant OTUs. Notably, although the number of OTUs decreased sharply, the number of reads clustered into these dominant OTUs decreased slightly in each sample (Table 1).

**Table 1:** Original and dominant OTUs and reads in the samples from injection well (IW) and production wells (PW1, PW2)

Sample name		IW	PW1	PW1T^a^	PW2	PW2T^a^
Original	OTU	282	259	346	165	219
	Reads	5070	5537	5099	4838	5763
Dominant	OTU	86	74	46	44	66
	Reads	4655	5200	4498	4618	5456
Coverage^b^ (%)		91.81	93.91	88.21	95.45	94.67

a “T” of PW1T and PW2T indicates the samples collected after the boost injection of nutrients.

b Coverage shows the percentage of the reads of dominant OTUs to the total number of valid reads detected.

### Comparing microbial communities inhabiting the injected water and the oil reservoir

The microbes usually present in the production waters collected from oil production wellheads are considered to be those inhabiting the oil reservoirs.^9,15^ Microbial communities detected in the injected water and the produced waters collected before the boost MEOR trial were compared to reveal the relationships between the microbes in the injected water and those in oil reservoirs which experienced the first round of MEOR treatment.^11^

**Fig. 2:**
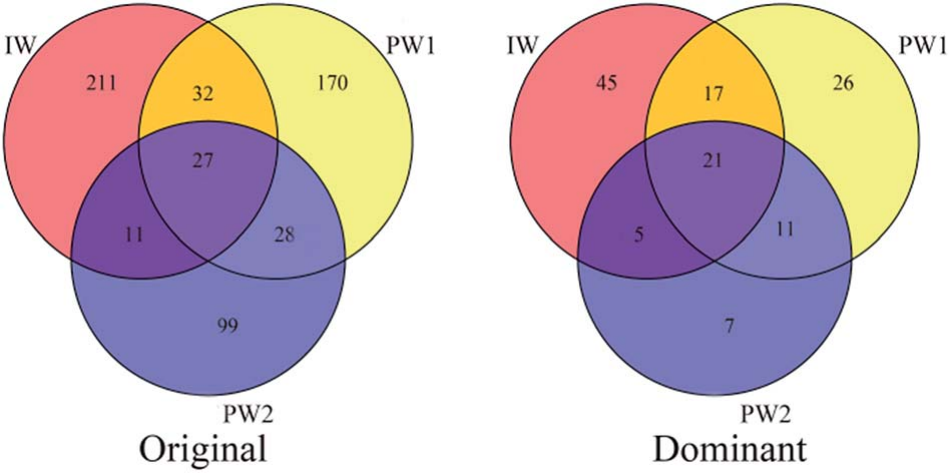
Shared OTU analysis of original OTUs and dominant OTUs for the samples. Venn diagram showing the unique and shared OTUs (3% distance level) in the different libraries of samples from injection well (IW) and the production wells of PW1 and PW2.

Shared OTUs can provide substantial data to show the relationships of microbial communities in different environments. In this study, shared OTU in Venn diagrams based on data showing the original OTUs and the dominant OTUs for the microbial populations of IW, PW1 and PW2 (Fig. 2) were analyzed. There were 70 shared OTUs which were detected in communities of the injected water and the produced waters, and significantly, a large percentage of these OTU (43 out of 70) were clustered into dominant OTUs. Whereas, small percentages of dominant OTUs were detected either only in the injected water or in the two produced waters (45 out of 211 OTUs and 44 out of 257, respectively).

**Fig. 3:**
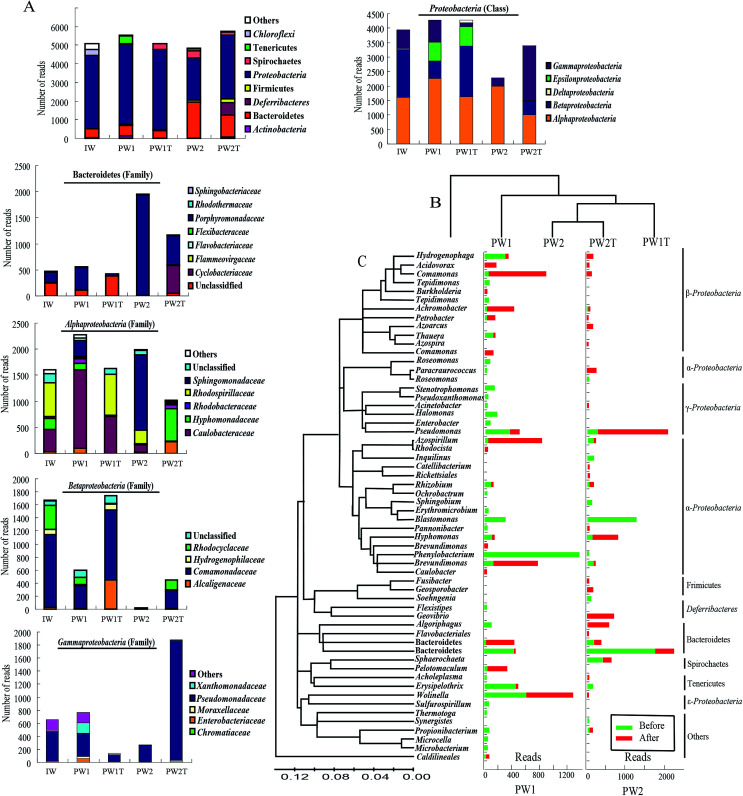
OTU alignment analysis of the samples from the injection well (IW) and the produced wells (PW1 and PW2) before and after the injection of boost nutrients in the oil reservoir. (A) Histograms showing the distributions of phyla in microbial communities of IW, PW1, PW1T, PW2, PW2T, and distribution of families present among the OTUs of the phylum Bacteroidetes and the three classes of the phylum Proteobacteria (Alphaproteobacteria, Betaproteobacteria and Gammaproteobacteria). (B) Samples and OTUs are clustered using their Bray–Curtis similarities (group-average linkage). (C) The bacterial phylogenetic tree of the dominant OTUs was calculated using the neighbor-joining method. Histograms showing the changes in numbers of reads of each OTU detected in samples collected from the produced wells (PW1 and PW2) before and after the injection of boost nutrients.

Based on the data of alignment, 23 different groups were identified within all the samples of the injection waters and the production waters. As a whole, the microbial communities within the three samples of IW, PW1 and PW2 after the first round of MEOR showed similar bacterial 16S rRNA distributions at the phylum level with slight variations in numbers (Fig. 3A). The populations of Proteobacteria were predominant in samples IW, PW1 and PW2 of the microbial communities and accounted for 77.81%, 77.33% and 44.17% of total reads, respectively. This was followed by populations of Bacteroidetes which represented 9.23%, 10.02% and 40.31%, in samples IW, PW1 and PW2, respectively. When reaching the class or family level, the differences of microbial community distributions increased gradually (Fig. 3A). Roughly, microbial groups were assigned dominantly into α-, β-, γ-Proteobacteria, and Porphyromonadaceae.

To further understand the relationship of important bacteria inhabiting injected water and the production waters, all the dominant OTUs were aligned to the level of genus and classified microbes into three groups present only in injected water, only in production waters and in both (Table 2). Those dominant only in injected water were affiliated to *Rhodovibrio* (12.01%), *Brachymonas* (11.38%), *Variovorax* (8.71%), *Azoarcus* (5.56%), uncultured *Chloroflexi* (4.99%) and *Sphingobacterium* (3.83%). In comparison the dominant reads detected only in produced waters were aligned to *Wolinella* (10.53% in PW1, 0.02% in PW2), *Erysipelothrix* (7.8% in PW1, 2.19% in PW2), *Blastomonas* (5.09% in PW1, 27.65% in PW2), *Halomonas* (2.82% in PW1), *Microbacterium* (1.21% in PW1), *Sphingobium* (2.51% in PW2) and *Soehngenia* (1.53% in PW2). Dominant groups appearing in both the injected water and the produced water belonged to *Pseudomonas*, uncultured Porphyromonadaceae, *Brevundimonas* and *Hyphomonas*. In this group, *Phenylobacterium* were dominant in PW1 with a percentage of 23.96%, whereas only a small percentage of reads (1.09%) of this genus was detected in the sample of the injected water.

**Table 2:** Dominant microbes inhabiting only the injected water, only the produced water or both the injected water and the produced water

Microorganisms detected								
Only in IW		Only in PW			In both IW and PW			
Microbes (genus)	Abundance^c^ (%)	Microbes (genus)	Abundance^c^ (%)		Microbes (genus)	Abundance^c^ (%)		
(OTU number)	IW		PW1	PW2		IW	PW1	PW2
*Rhodovibrio*	12.01	*Wolinella*	10.53	0.02	***Pseudomonas*** a	8.32	6.06	5.04
*Brachymonas*	11.38	***Erysipelothrix*** b	7.8	2.19	***Brevundimonas*** a	6.92	2.28	2.54
*Variovorax*	8.71	***Blastomonas*** b	5.09	27.6	***Hyphomonas*** a	4.02	2.13	0.06
***Azoarcus*** a	5.56	**Spirochaetes** b	0.04	7.46	Uncultured Porphyromonadaceae	3.75	7.13	39.1
Uncultured Chloroflexi	4.99	*Pannonibacter*	0.51	0.02	***Azospirillum*** a	0.18	0.41	2.25
*Sphingobacterium*	3.83	***Halomonas*** b	2.82	0	*Roseomonas*	0.5	1.37	0.52
Uncultured Alcanivoracaceae	1.24	Uncultured Bacteroidia	1.63	0	*Stenotrophomonas*	0.02	2.81	0.02
Uncultured Ectothiorhodospiraceae	1.14	*Microbacterium*	0.6	0	*Enterobacter*	0.12	1.07	0.04
*Opitutus*	1.04	*Sulfurospirillum*	0.92	0	*Rhizobium*	0.1	1.44	0.47
*Methylocystis*	0.99	***Tepidimonas*** b	0.74	0	**Uncultured Bacteroidales** a	0.28	0.29	0.1
Uncultured Caldilineaceae	0.91	***Pseudoxanthomonas*** b	0.45	0	***Comamonas*** a	0.32	0.81	0.04
Uncultured *Rhizobiales*	0.81	*Microcella*	0.59	0	***Achromobacter*** a	0.04	0.2	0.17
Uncultured Gemmatimonadetes	0.73	*Flexistipes*	0.43	0	***Petrobacter*** a	1.12	0.31	0.08
*Parvibaculum*	0.69	***Acinetobacter*** b	0.42	0	*Propionibacterium*	0.18	0.99	0.19
Uncultured Burkholderiales	0.65	*Ochrobactrum*	0.36		***Phenylobacterium*** b	1.09	23.96	0
*Legionella*	0.49	*Pelotomaculum*	0.31	0	***Hydrogenophaga*** b	0.88	5.2	0
*Thiobacillus*	0.46	*Thermotoga*	0.25	0	*Acidovorax*	0.02	0.06	0
*Alcaligenes*	0.41	*Sphingobium*	0	2.15	*Tepidimonas*	0.18	1.81	0
*Holosporaceae*	0.36	*Soehngenia*	0	1.53	*Thauera*	1.22	1.86	0
*Altererythrobacter*	0.32	*Synergistes*	0	0.25	***Sphaerochaeta*** a	0.24	0.34	0
Uncultured Solibacteraceae	0.32				*Planctomyces*	1.95	0.04	0

a Bold microbe names are those that were activated.

b Bold microbe names are those that were inhibited.

c Abundance showing the relative abundances calculated based on the numbers of reads.

### Microbial community responses to the MEOR field trial application

Injection of nutrients would inevitably result in an alteration of microbial communities in oil reservoirs, which would be revealed by the dynamics of microbial communities in samples collected from the same production well before and after injection of nutrients. In this research, firstly, the hierarchical cluster analysis gave a preliminary insight into the relationships of the microbial communities in the samples, revealed by the fact that PW2 and PW2T microbial communities were clustered together, and then grouped with the PWIT, PW1 and IW (Fig. 3B). The number of detected OTUs within both samples of produced waters increased slightly after the boost MEOR field trial.

To gain in-depth insight into the microbes that were stimulated by the boost MEOR process, the microbe of each dominant OTU was analyzed (Fig. 3C). Nutrient injection during the boost MEOR field trial resulted in an obvious increase in relative abundance of some OTUs (Fig. 3C). In the samples from the production well, J70-050C (PW1), the relative abundance of the OTU was associated closely with the sharp increase of *Comamonas* from 0.79% (44) to 15.3% (780) after nutrient injection. This was followed by the OTU associated with *Brevundimonas* with an increase in relative abundance from 0.31% (17) to 11.83% (603), and the relative abundance of the OTU clustered within *Azospirillum* increased from 0.34% (19) to 11.79% (610). Similarly the relative abundance of the OTU grouped into *Achromobacter* increased from 0.2% (11) to 7.4% (359) whereas those for the OTU of the uncultured Bacteroidales increased from 0.27% (15) to 6.2% (316) and for OTU of *Sphaerochaeta* abundance increased from 0.34% (19) to 5.68% (269).

In comparison, in the samples from the production well, J68-552 (PW2), the most visible increase in relative abundance after nutrient injection occurred for the OTU associated with *Pseudomonas*, which increased from 2.25% (109) to 26.24% (1512), followed by the OTU aligned to *Hyphomonas* which increased from 0.06% (3) to 10.36% (597). Surprisingly, in the sample collected from the production well, J68-552 (PW2) before the injection of boost nutrients, not a single read of six OTUs was detected, whereas, microbes aligned to the six OTUs were dominant in the sample collected after the injection of nutrients (PW2T). These six OTUs were associated with *Geovibrio* (646, 11.21%), *Algoriphagus* (524, 9.09%), *Paracraurococcus* (216, 3.75%), *Hydrogenophaga* (135, 2.34%), *Azoarcus* (124, 2.15%) and *Geosporobacter* (124, 2.15%). Among these OTUs, however, reads of OTUs affiliated with *Algoriphagus*, *Paracraurococcus*, *Hydrogenophaga* and *Azoarcus* appeared in the samples of the injection well (IW) and the production well, J70-50C (PW1).

From Fig. 3C, it can also be observed that there is a decrease in relative abundance of some OTUs after the injection of boost nutrients. In the J70-50C (PW1) well, not a single read of the OTU affiliated with *Phenylobacterium* was detected in the sample PW1T. Whereas, *Phenylobacterium* was dominant with a percentage of 23.01% (1274, reads) in the sample PW1. This was followed by the OTU associated with *Blastomonas*, *Halomonas*, *Pseudomonas*, and uncultured Porphyromonadaceae, which were present at a relative abundance of 4.75%, 2.82%, 4.73% and 2.19%, respectively, in PW1. In addition, the relative abundance of the OTU associated to *Erysipelothrix* decreased significantly from 7.46% (413) to 0.22% (11). In the J68-552 (PW2) well, not a single read of the OTUs associated with *Blastomonas* was detected after nutrient injection, whereas, this genus was dominant in well PW2 with a relative abundance of 25.42% (1230). There was a visible decrease of relative abundance shown in the OTU associated to uncultured Porphyromonadaceae with a drop from 35.78% (1731) to 7.69% (443), and in Spirochaetes with a decrease from 7.46% (361) to 3.05% (176).

In this study, only members belonging to the genus of *Wolinella* seemed to be not affected by the injection of nutrients. Reads of OTU aligned to *Wolinella* remained almost similar at 583 (10.53%) and 648 (12.71%) in PW1 and PW1T, respectively. Numbers belonging to this genus were few in sample PW2 and also had no significant change in numbers after the injection of nutrients.

## Discussion

### The relationships between microbial groups present in the injection water and those in MEOR treated oil reservoirs

Active microbes in the injection water or in the oil reservoirs would establish new microbial populations after the first round of nutrient injection, which would reflect the present microbial ecology associated with the present oil reservoirs.^15^ The relationships of microbes inhabiting the water normally injected into the first round MEOR processed oil reservoir and those present in the samples collected from the production wells were analyzed to reveal what organisms predominated in the present oil reservoir.

The detected OTUs present showed that there were differences in microbial community in the injected water and the produced waters. However, 43 were shared OTUs, representing half of dominant OTUs in each microbial community of IW, PW1 and PW2 (Fig. 2), which indicated the relationships of the resident microbial populations in the injected water and the produced water.

Generally, most of the microbes in the injected water and produced water were mainly clustered into two phyla Proteobacteria and Bacteroidetes. Such an observation was similar to many published reports of microbial populations inhabiting oil reservoirs.^3–7^ However, at the level of genus, different groups of microbes were detected in the injected water and the produced water.

The dominant groups of microbes which were only detected in the produced water were associated with *Wolinella*, *Erysipelothrix*, *Blastomonas*, Spirochaetes, *Halomonas*, *Sphingobium* and *Soehngenia*. In general, microbes detected in the produced waters showed some special characteristics in metabolism and had a close similarity to those detected in the special environment of oil reservoirs because there are common characteristics of oil reservoirs such as having limited electron acceptors and donors and a large amount of hydrocarbons.^2,21^ Consistently, the sequence affiliated with *Wolinella* showed the closest similarity with the clone 38TB (GQ37747) detected in the anaerobic phenol degrading enrichment cultures.^22^ The sequence of *Sphingobium* was closest to the clone BZ13 (HQ58831, unpublished) isolated from hydrocarbon contaminated soil. Furthermore, the sequences of *Erysipelothrix*, Spirochaetes, *Soehngenia* were associated most closely to the clones [PL-6B11 (AY570636), LHJB-126 (JF741946) (unpublished), clone NK-11 (JN685469), respectively] detected in samples from oil reservoirs.^20,23,24^ It is of interest to note that most of these microbes had anaerobic characteristics. In addition, sequences of *Blastomonas* and *Halomonas*, not affiliated with microbes associated with hydrocarbon degradation nor detected in oil reservoirs, but showed a close similarity with microbes detected in environments with water,^25,26^ which suggests that the origin of these microbes were from the local water resources such as formation water.

It should be noticed that the proportion of some microorganisms detected in PW1 and PW2 showed a great difference (Table 2). The causes for this may be the heterogeneity of the oil reservoir and the differences of microbe distribution between the different layers.

The dominant microbes only detected in the injected water were associated with the genera *Rhodovibrio*, *Brachymonas*, *Variovorax*, *Azoarcus*, *Sphingobacterium*, and *Opitutus*. Although there was not a single sequence of which showed close similarity with those regularly detected in oil reservoirs, sequences of *Brachymonas*, *Variovorax*, *Azoarcus* and *Opitutus* were aligned closest to microbes detected in a wastewater plant of a petroleum refinery [strain CXH (AY275432), unpublished], polycyclic aromatic hydrocarbons (PAH) polluted soil [clone PL58 (FR853224), unpublished], aerobic activated sludge found during different produced water treatments [clone HB77 (EF648075)], and contaminated soil from an oil field [clone 2-54 (KC521825)], respectively. In addition, the sequences of *Rhodovibrio* were close to microbes detected in hypersaline wastewater [clone MU035 (AM157601)].^27^ These results of alignment also gave an impression that a major number of microbes detected in injected waters were associated with aerobic removal of hydrocarbons.

Dominant microbes which appeared in both the injected water and the produced water included the genera of *Pseudomonas*, *Brevundimonas*, *Hyphomonas* and uncultured Porphyromonadaceae. These microbes seemed to possess the ability to survive in environments of both the surface and the subsurface. Microbes associated with *Pseudomonas* were never considered be indigenous to oil reservoirs.^2^ Remarkably though, members of this genus were often detected in the samples from oil reservoirs.^3,5,6^ The capacity of lateral gene transfer possessed by the genus of *Pseudomonas* might support the survival and diversity after introduction into oil reservoirs.^28^ This genus of microbes inhabiting oil reservoirs were identified as hydrocarbon degraders which were occasionally capable of biosurfactant production.^13,29,30^ The sequence of *Brevundimonas* was closest to the clone MW-B01 (JQ088317) detected in a long-term water flooded oil reservoir^24^ and this genus has been described as an oil degrader.^21,31^ Sequences of *Hyphomonas* and uncultured Porphyromonadaceae were also detected in samples from oil reservoirs and the environment affected by prestige oil spill contamination.^4,9,32^

In a summary, a close microbial community compositional relationship between the injection water and the successful MEOR processed oil reservoir was revealed using 454 pyrosequencing and bioinformatic analysis. A number of shared dominate OTUs (43) were determined between the microbial communities of injection water and production water collected from the first round nutrient injection oil reservoir. The main reason for this may be that the microbes inhabiting the injection water or in the reservoir, reacted to the injected nutrients and would grow up fast, and then some might flow out with crude oil and water, persisting in the recycled injection water that separated from the produced fluid, so the connection was created.

### Nutrient injection effects on microbial communities occurrence

When nutrients are injected to the oil reservoirs, the process can be monitored by the increase in number of certain microbes, the loss of substrate, and the appearance of specific products of metabolism based on culture dependent and culture independent methods.^2,33^ Alterations in the microbial community during a MEOR definitely showed a whole response of the resident microbes to injection of nutrients. In this study, the main increase in the relative abundance occurred in shared microbes, detected both in the injected water and the produced waters. Furthermore, after boost nutrients were injected, microbes of the genera *Erysipelothrix* and *Blastomonas*, dominant and detected only in the produced waters, were inhibited. In addition, members of *Phenylobacterium* that were predominant in the produced well of J70-50C (PW1) were also inhibited (Table 1), which might reveal a potential pattern that microbes inhabiting both injected water and the produced waters were more competitive to nutrients injected in the oil reservoirs during a MEOR than those that only survive in oil reservoirs.

In this study, the activated microbes in the produced wells belonged to the genera of *Comamonas*, *Achromobacter*, *Azospirillum*, *Brevundimonas*, uncultured Bacteroidales, *Sphaerochaeta*, *Paracraurococcus*, *Pseudomonas* and *Hyphomonas*. Notably, there were two groups of *Geovibrio* and *Algoriphagus* that were too few to be detected before the injection of boost nutrients that were also activated. Without further isolation and metabolic analysis, it was not possible to determine specifically which microbial activities took place and which products were produced during the process of MEOR by the activated microbes. To a lesser extent, microbial mechanisms for oil recovery act synergistically in the subsurface environments.^2^ Because of the complexity and uncertainty of the MEOR field trial, and given the fact that the MEOR field trial described in this paper was on a small scale, so the sample repetition in this research was insufficient. However, microbial dynamics analysis still could provide an indication for the alterations of various microbes in the MEOR process, which is of great importance in guiding further studies to determine the potential application of the microbes.

In summary, it has been concluded that based on the shared resident OTUs microbes in the oil reservoirs and in the injected water had close relationships. Microbes detected in the present study showed the closest similarities with those detected in samples from oil reservoirs or those associated with hydrocarbon degradation, which displays a picture of microbes that were special to the microbial ecology of oil reservoirs. After nutrients were injected, microbes that survived both in the injected water and in the oil reservoir seemed to be easily activated. The microbial communities present only in, or dominant within the produced waters suffered inhibition with a sharp decrease in relative abundance, which provided a potential pattern for preferential selection of microbes for MEOR stimulated *in situ*. It is worth noting that further research based on culture dependent methods is necessary to confirm the potential activities or products possessed by the activated microbes for MEOR.

## Conclusions

Based on 43 shared dominant operational taxonomic units (OTUs) detected both in the injection water and the production water, only those microbes co-existing in the injection water and the production water gave positive responses after the injection of boost nutrients. It was concluded that there was a close microbial community compositional relationship between the injection water and the successful MEOR processed oil reservoir and it is proposed that the nutrient requirement should be re-optimized, rather than just using simple repeats of the injection of nutrients for the subsequent *in situ* MEOR application in that reservoir.

## Conflicts of interest

There are no conflicts to declare.

## Supplementary Material
